# Novel Lipoprotein (a) Therapies: A Comprehensive Review

**DOI:** 10.1007/s11883-026-01453-9

**Published:** 2026-08-13

**Authors:** Ugochukwu Ebubechukwu, Onyinye Ugoala, Rameen Shahid, Anandita Kulkarni

**Affiliations:** 1 Department of Medicine, SUNY Downstate Health Sciences University, Brooklyn, NY USA; 2 Division of Cardiovascular Medicine, The University of Texas Health Science Center at Houston, Houston, TX USA; 3 Department of Internal Medicine, Texas Tech University Health Sciences Center, Amarillo, TX USA; 4 Department of Internal Medicine, Hunt Regional Medical Center, Greenville, TX USA; 5 Center for Cardiovascular Disease Prevention, Baylor Scott & White The Heart Hospital-Plano, Plano, TX USA

**Keywords:** Lp(a), Atherosclerotic cardiovascular disease, Antisense Oligonucleotides (ASOs), Small Interfering RNAs (siRNAs), Muvalaplin, CTX320

## Abstract

**Purpose of Review:**

Lipoprotein(a) [Lp(a)] is a genetically determined, independent risk factor for atherosclerotic cardiovascular disease and calcific aortic valve stenosis. Plasma Lp(a) levels are 70–90% heritable, largely unresponsive to lifestyle changes, and poorly controlled with typical lipid-lowering drugs such as statins and PCSK9 inhibitors. The development of powerful RNA-based therapies offers a new chance to specifically target and significantly lower Lp(a). We examined available phase 1–3 trial data, regulatory updates, and trial registries up to mid-2026 to provide an overview of the current pipeline, ongoing cardiovascular outcomes trials (CVOTs), and new mechanistic and genomic methods.

**Recent Findings:**

Five agents across three mechanisms have reached late-stage development. Pelacarsen, a GalNAc-conjugated antisense oligonucleotide (ASO), reduces Lp(a) by about 80% via hepatic apo(a) mRNA reduction and is the subject of the phase 3 Lp(a) HORIZON trial, with results due in late 2026. Three small interfering RNA (siRNA) drugs, olpasiran (> 95% reduction, OCEAN(a)-Outcomes), lepodisiran (93.9% reduction lasting over 12 months after one dose, ACCLAIM-Lp(a)), and zerlasiran (96.4% reduction), allow quarterly or possibly yearly dosing. Muvalaplin, the first oral small-molecule Lp(a) inhibitor, interferes with apo(a)-ApoB particle formation and reduces intact Lp(a) by up to 85.8% in phase 2 KRAKEN trials; its phase 3 CVOT (MOVE-Lp(a)) is underway. Additionally, CRISPR/Cas9-based liver gene editing targeting the LPA gene (CTX320) has entered phase 1 studies. All agents have shown good safety so far.

**Summary:**

The Lp(a) treatment landscape has moved from basic science to an active late-stage clinical pipeline. Positive outcomes from these CVOTs could lead to regulatory approvals and guideline updates affecting hundreds of millions of high-risk patients globally. Recent guidelines now recommend universal Lp(a) measurement, in anticipation of more effective therapies becoming available within the next 1 to 2 years.

**Graphical Abstract:**

**Figure:**
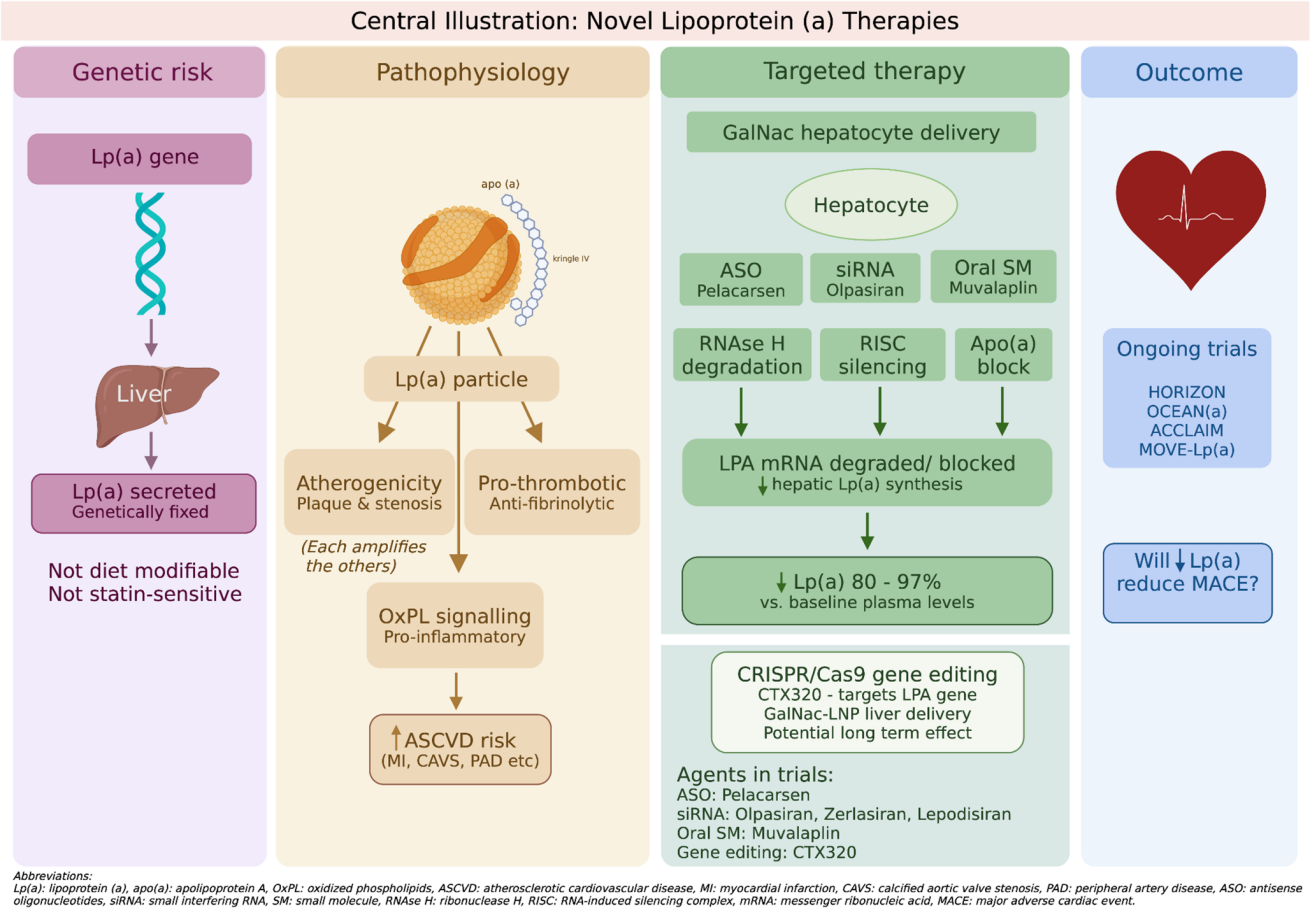
Created in BioRender. https://biorender.com/y1mu0lh

## Introduction

Lipoprotein (a) [Lp(a)] has been established as an independent, genetically determined causal risk factor for atherosclerotic vascular disease (ASCVD), calcific aortic stenosis, and other cardiovascular conditions [1, 2]. Lp(a) is a structurally unique macromolecule composed of apolipoprotein B-100 (ApoB) covalently linked to apolipoprotein (a) encoded by the LPA gene on chromosome 6q26-27, enriched with oxidized phospholipids that confer atherogenic, thrombotic, and pro-inflammatory properties [3].

Lp(a) levels are approximately 70% to > 90% genetically determined and individuals with Lp(a) levels < 75 nmol/L (30 mg/dL) are considered low risk, individuals with Lp(a) levels ≥ 125 nmol/L (50 mg/dL) are considered high risk, and individuals with Lp(a) levels between 75 and 125 nmol/L (30–50 mg/dL) are at intermediate risk [1, 4]. Elevated Lp(a) (> 100–125 nmol/L, ∼50 mg/dL) levels affect approximately 20% of the global population [5]. Nordestgaard and Langsted reported that lipoprotein(a) concentrations are lowest among individuals from East Asia, Europe, and Southeast Asia; intermediate among those from South Asia, the Middle East, and Latin America; and highest among individuals from Africa [6]. Because Lp(a) levels are genetically determined, unlike LDL cholesterol (LDL-C), they are largely unresponsive to lifestyle modifications, such as dietary changes, or conventional lipid-lowering therapies, such as statins (a major cornerstone in ASCVD prevention) [7].

Despite the establishment of Lp(a) as a strong independent risk factor for ASCVD, current therapeutic options remain limited. However, the recent era has seen significant pharmacological innovations with five investigational agents in advanced clinical development with multiple landmark cardiovascular outcomes trials either completed or ongoing, and the realistic prospect of the first approved Lp(a)-lowering therapy currently scheduled within the next 1–2 years.

### Mechanistic Relationship Between Elevated Lp(a) and Cardiovascular Disease

Lp(a) drives ASCVD primarily through three interconnected pathways, each amplifying the others, namely: atherogenicity, pro-thrombotic/anti-fibrinolytic, and pro-inflammatory signaling via oxidized phospholipids (OxPLs) [8, 9]. Lp(a), via its OxPL component, increases the expression of endothelial adhesion molecules and cytokines, promoting monocyte transmigration in vitro [1]. Lp(a) also accumulates in the arterial intima more readily than LDL, partly because apo(a)’s structural features improve its binding to intimal proteoglycans [10]. Once deposited, it stimulates foam cell formation, smooth muscle cell proliferation, and plaque growth [10]. Lp(a) promotes blood clot formation and inhibits clot breakdown through its component apo(a), which resembles plasminogen, the precursor that breaks down fibrin clots [1]. Since apo(a) competes with plasminogen for binding to fibrin and cell-surface receptors, it hinders fibrinolysis without itself becoming an active enzyme [1]. Consequently, high levels of Lp(a) shift the hemostatic balance, favoring clot stability. Lp(a) also primarily carries OxPLs in plasma [1, 11, 12]. These bioactive lipids, created through the oxidative modification of phosphatidylcholine on Lp(a)’s surface, activate pattern recognition receptors on endothelial cells and monocytes, leading to adhesion molecule expression, cytokine secretion, and a boost in inflammatory responses [11]. In the aortic valve, OxPLs promote calcification by inducing osteoblastic transdifferentiation of valve interstitial cells, which explains why Lp(a) is more strongly linked to calcific aortic valve stenosis than LDL, even though LDL plays a larger role in coronary disease [1, 13, 14].

These three interconnected mechanisms show that Lp(a)-rich plaques are more lipid-heavy, inflamed, and prone to thrombosis. When such a plaque ruptures, impaired fibrinolysis leads to larger, more persistent thrombi, and the OxPL burden exacerbates inflammation [10]. This explains why Mendelian studies show that the cardiovascular risk attributable to Lp(a) is disproportionate to its cholesterol content alone, and why it remains a risk even in patients with well-managed LDL-C on maximal statin therapy [6, 15].

### Pharmacological Management of Elevated Lp (a) Levels

Several pharmacological agents are currently in different development phases aimed at lowering high Lp(a) levels. Several large clinical trials are ongoing to assess their safety, tolerability, and effectiveness. While some new therapies are still being researched, existing lipid-lowering treatments have been evaluated for their impact on reducing Lp(a). These studies show mixed, often modest results, underscoring the urgent need for more targeted treatment options.

#### Current Lipid-Lowering Therapies

##### Statins

Statins have been established as the gold standard for lipid-lowering agents. Statins remain the cornerstone of LDL-C-lowering therapy, and all major guidelines recommend intensifying LDL-C lowering, including with statins, in patients with elevated Lp(a), given the well-established cardiovascular benefit of LDL-C reduction irrespective of Lp(a) status. However, statins do not lower Lp(a) itself; evidence instead suggests that statins may increase Lp(a) levels by roughly 10–20% [16]. Also, evidence from a meta-analysis suggests that statins do not improve Lp(a) plasma levels or reduce the CVD risk related to elevated Lp(a) levels [17]. However, a pooled individual patient-data meta-analysis of seven randomized, placebo-controlled statin outcome trials (4D, 4 S, FLARE, JUPITER, LIPID, MIRACL, and TNT) found no significant overall change in Lp(a) levels with statin therapy, indicating that the modest elevations reported in smaller studies do not represent a consistent, clinically significant effect [18]. 

##### Niacin

Niacin has been used over the years at a recommended dose of 1–3 g per day to manage dyslipidemia when combined with statins. Data emerging over the past few years have raised concerns about the efficacy of niacin as an effective pharmacological agent in reducing CVD risk [19]. Outcome trials (AIM-HIGH and HPS2-THRIVE) showed no incremental cardiovascular benefit and significant side effects (flushing, liver toxicity, new-onset diabetes, myopathy) [20, 21]. D’Andrea et al., in their meta-analysis and systematic review of 119 clinical trials, demonstrated that although extended-release niacin is currently used as monotherapy for dyslipidemia, its indications are based on findings from older studies and may not align with contemporary management guidelines [22].

##### PCSK9 Inhibitors

Proprotein convertase subtilisin/kexin type 9 (PCSK9) is a protein that binds to specific regions of LDL receptors, forming a complex that, when internalized, leads to lysosomal degradation of the LDLR and reduces the clearance of LDL-C from the circulation [19]. PCSK9 inhibitors, such as evolocumab and alirocumab, are monoclonal antibodies that inhibit PCSK9 activity, thereby reducing circulating LDL-C levels [19]. In the ODYSSEY OUTCOMES trial, alirocumab decreased Lp(a) by a median of 5 mg/dL, and every 5 mg/dL reduction in Lp(a) was associated with a 2.5% decrease in CVD risk [23]. While in the FOURIER trial, evolocumab reduced Lp(a) levels by a median of 26.9% [24].

##### Inclisiran

Small interfering RNA (siRNA) uses nucleotide base-pairing complementarity to bind to and inhibit the translation of specific messenger RNA (mRNA) sequences, thereby silencing gene expression, and would be discussed further in a later part of this review. Inclisiran is a siRNA that acts by attenuating the hepatic synthesis of PCSK9. This inhibition of PCSK9 production primarily leads to an increase in LDLR on the hepatocellular cell membrane and subsequently enhanced clearance of LDL-C [25]. The reduction in Lp(a) is a concomitant beneficial effect of inclisiran, as demonstrated in several ORION trials [25]. The ORION-1 trial reported reductions of 15% to 26% in Lp(a) levels, while the ORION-9, -10, and − 11 trials, in which inclisiran decreased Lp(a) levels by 13.5%, 21.9%, and 18.6% from baseline to day 540, respectively [25]. Also, a prespecified analysis of the ORION-11 trial showed a marked reduction in Lp(a) levels by 28.5% at day 540 [25].

##### Lipoprotein Apheresis

Involves extracorporeal removal, achieving a reduction in Lp(a) levels by 60–75%, and is recommended in high-risk patients with progressive cardiovascular disease despite maximal medical lipid-lowering therapy [26, 27]. It is important to note that the FDA has not approved any pharmacological interventions to date to lower Lp(a) levels, including those mentioned above; however, lipoprotein apheresis may be used in selected high-risk patients [28, 29]. Some major considerations include transient effects (levels usually rebound within 1–2 weeks), procedure burden, and limited access.

### Current Drugs in Development

The newer therapies to lower Lp(a) include nucleic acid-based drugs, such as antisense oligonucleotides (ASOs) and siRNA, which offer a promising strategy for targeting the LPA transcript in hepatocytes and potently suppressing Lp(a) production [30] (Table 1).

**Table 1:** The major ongoing Lp(a)-lowering therapy cardiovascular outcomes trial landscape

Trial	Drug	Number enrolled	Lp(a) threshold	Population	Primary endpoint	Estimated readout
Lp(a) HORIZON	Pelacarsen	8,323	≥ 175 nmol/L (≥ 70 mg/dL)	2° prevention (ASCVD)	4-pt MACE	Mid-Late 2026
OCEAN(a)-Outcomes	Olpasiran	~ 7,297	≥ 200 nmol/L	2° prevention (ASCVD)	CHD death/MI/ revascularisation	Late 2026/2027
ACCLAIM-Lp(a)	Lepodisiran	17,300 (target)	≥ 175 nmol/L	2° prevention (ASCVD) + high-risk 1° prevention	4-pt MACE	2029
MOVE-Lp (a)	Muvalaplin	10,450 (target)	≥ 175 nmol/L	2° prevention (ASCVD) + high-risk 1° prevention	MACE	2031
OCEAN(a)-PreEvent	Olpasiran	11,000 (target)	≥ 200 nmol/L	1° prevention	MACE	2031

*Lp(a) *Lipoprotein (a), *ASCVD *Atherosclerotic cardiovascular disease, *MACE *Major adverse cardiovascular events, *CHD *Coronary heart disease

#### Antisense Oligonucleotides

Antisense oligonucleotides (ASOs) are synthetic single-stranded oligonucleotides with up to 30 bases in length that target and bind to specific RNAs. They hinder translation by blocking, degrading, or, more commonly, cleaving via the ribonuclease H (RNase H) mechanism, acting on Watson-Crick complementarity.

Pelacarsen (formerly known as AKCEA-Apo(a)-LRx or TQJ230 or ISIS 681257 or IONIS-Apo(a)-LRx) represents a breakthrough in this field. Pelacarsen is an example of a second-generation ASO [19]. It features a trifurcated N-acetylgalactosamine (GalNAc) molecule chemically attached to it [19]. The complex is rapidly taken up by the asialoglycoprotein receptor (ASGPR), which is abundant on hepatocytes, the same cells where Lp(a) is produced [19]. Pelacarsen enters the hepatocyte nucleus and binds specifically to the mRNA that encodes apolipoprotein(a) [apo(a)], forming an mRNA-ASO duplex that is selectively cleaved by ribonuclease H (RNase H), thereby blocking apo(a) production and reducing Lp(a) levels [31].

Randomized trials have demonstrated the efficacy of ASOs in markedly reducing plasma Lp(a) levels [19]. Tsimikas et al. conducted a phase 2 double-blind dose-ranging RCT involving 286 patients with established CVD and elevated Lp(a) levels of at least 60 mg/dL [32]. These patients were assigned to receive pelacarsen 20, 40, or 60 mg every 4 weeks; 20 mg every 2 weeks; 20 mg every week; or placebo subcutaneously for 6 to 12 months [32]. Pelacarsen was associated with a dose-dependent reduction in plasma Lp(a) levels, with mean decreases ranging from 35% to 80% across doses, compared with a 6% reduction in the placebo group (p values ranged from 0.003 to < 0.001) [32]. Among patients in the highest dose group, 98% achieved Lp(a) levels ≤ 50 mg/dL, and 71% achieved levels ≤ 30 mg/dL [32].

An ongoing phase 3 clinical trial, NCT04023552, is assessing the impact of lipoprotein(a) lowering with Pelacarsen (TQJ230) on major cardiovascular events in patients with established CVD (Lp(a)HORIZON) and is expected to be completed in 2026 [33]. Additional ongoing pelacarsen studies include the Lp(a)FRONTIERS CAVS (NCT05646381), a phase 2 study assessing pelacarsen’s impact on progression of calcific aortic valve stenosis in patients with mild-to-moderate CAVS and elevated Lp(a); Lp(a)FRONTIERS PEARL (NCT07625306), a new phase 3b study (not yet recruiting as of May 2026) evaluating initiation of pelacarsen in US patients with recent ACS (STEMI/NSTEMI) and elevated Lp(a), with a coronary plaque sub-study; estimated start August 2026; and a phase 3b diversity study (NCT06267560), specifically in US Black/African American and Hispanic patients with elevated Lp(a) and established ASCVD (primary completion March 2026), addressing critical equity gaps in representation across Lp(a) trials [34–36].

#### Small Interfering RNA (siRNAs)

siRNAs are usually duplex RNA molecules 21–23 nucleotides long, comprising a passenger (sense) strand and a guide (antisense) strand. It is important for their length to be less than 30 nucleotides; otherwise, siRNAs may be recognized as antigens by innate immune receptors, such as toll-like receptors. siRNA consists of a guide strand that binds to the target mRNA and a passenger strand [37]. Within the cell, it becomes part of the RNA-induced silencing complex (RISC) [37]. The passenger strand is released, allowing the active RISC to cleave the target mRNA when the guide strand pairs with its target sequence [37]. siRNA offers a unique gene-silencing mechanism, and recent advances in siRNA technology have enabled safe, potent, and durable target suppression.

Olpasiran (formerly AMG 890 or ARO-LPA), developed by Amgen, is a synthetic, N-acetylgalactosamine (GalNAc)-conjugated (hepatocyte-targeted) siRNA targeting LPA, modified with 2′-fluoro and 2′-methoxy substitutions, as well as phosphorothioate internucleotide linkages at the termini to help stabilize the duplex [30]. Olpasiran is loaded into RISC, which repeatedly cleaves apo(a) mRNA, enabling less frequent dosing than pelacarsen [38]. Koren et al. demonstrated that, from an initial set of 108 siRNA sequences targeting human LPA, selected using a bioinformatics algorithm, Olpasiran emerged as the lead candidate for clinical testing [30]. This phase 1 study (NCT03626662) was conducted at eight centers, and showed Lp(a) reduction occurring in a dose-responsive manner, with a maximum mean percent change from baseline ranging from − 71% to − 97% in cohorts 1–5 and from − 76% to − 91% in cohorts 6 and 7 [30]. The maximum absolute change in Lp(a) ranged from − 85 nmol/L to − 140 nmol/L in cohorts 1–5 and from − 198 nmol/L to − 266nmol/L in cohorts 6 and 7 [30]. Based on the aforementioned clinical trial, another phase 1 randomized parallel-group study was conducted, involving both healthy Japanese and non-Japanese subjects (27 in total) [39]. The study evaluated the effects of olpasiran at single ascending doses in the Japanese subjects and at a fixed dose of 75 mg in non-Japanese subjects. There was a dose-proportional reduction in Lp(a) levels from baseline, ranging from 56% to 99% [39]. These reductions were noted as early as day 4 after administration. Notably, the effect on Lp(a) did not differ significantly between Japanese and non-Japanese subjects [39].

Among the 281 enrolled patients in the OCEAN (a)-DOSE trial with a mean baseline of approximately 260 nmol/L, olpasiran therapy significantly reduced the lipoprotein(a) levels in a dose-dependent manner, with placebo-adjusted mean percent changes of − 70.5% with the 10-mg dose, − 97.4% with the 75-mg dose, − 101.1% with the 225-mg dose administered every 12 weeks, and − 100.5% with the 225-mg dose administered every 24 weeks (*P* < 0.001 for all comparisons with baseline) [40]. Phase 2 OCEAN(a)-DOSE data demonstrated nearly complete suppression of Lp(a), with placebo-adjusted reductions surpassing 100% at the 225 mg dose [40]. During the extension period, Lp(a) levels stayed about 40–50% below baseline, even nearly a year after discontinuing therapy [41]. This notable off-treatment persistence, not observed with pelacarsen, likely results from differences in the kinetics of RISC-mediated versus RNase H-mediated mRNA silencing. Additionally, olpasiran significantly decreased OxPL-apoB (an indicator of OxPL cargo), with about a 51.6% reduction at week 36 in the OCEAN(a)-DOSE trial, even though it did not notably lower hs-CRP or IL-6 [42]. This indicates that reducing OxPL could offer anti-atherogenic benefits beyond just decreasing Lp(a) mass, a finding with important mechanistic implications.

The ongoing (OCEAN(a))-Outcomes, phase 3 clinical trial, NCT05581303, compares the effect of treatment with olpasiran to placebo on the risk for coronary heart disease death, myocardial infarction, or urgent coronary revascularization in participants with known atherosclerotic cardiovascular disease and elevated Lp(a) [43]. OCEAN(a)-Outcomes Trial is estimated to be completed in December 2026, with results anticipated in 2027 [43]. Ongoing trials of olpasiran include OCEAN(a)-CCTA (NCT07293260), a recently initiated randomized study expected to start in March 2026, which assesses the impact of olpasiran on coronary artery plaque burden using coronary CT angiography [44]. Additionally, the OCEAN(a)-PreEvent (NCT07136012), launched in August 2025 and currently recruiting, is a landmark phase 3 trial investigating olpasiran for primary prevention in patients with elevated Lp(a) but no prior cardiovascular events [45]. Expected to conclude in October 2031, this trial is critically important: most patients with high Lp(a) are identified before any cardiovascular event, and its results will determine if prophylactic Lp(a) reduction is justified.

Zerlasiran (formerly SLN360) is another siRNA conjugate with a trifurcated GalNAc molecule that targets and inhibits the translation of LPA mRNA into apo(a) in hepatocytes [46]. SLN360 significantly reduced LPA mRNA molecules in primary human hepatocytes and in healthy cynomolgus monkeys, resulting in a serum Lp(a) reduction of over 95% from baseline for at least 9 weeks after dosing [46]. In the first phase 1 study (APOLLO trial) involving 32 healthy participants with elevated serum Lp(a) levels (more than 70 mg/dL), the safety, tolerability, pharmacokinetics, and pharmacodynamics of the medication were assessed [47]. Participants were randomly assigned to receive single ascending doses of SLN360 (30, 100, 300, and 600 mg) or placebo, with the primary outcome being the evaluation of safety, tolerability, and the effect on serum Lp(a) levels on day 150 [47]. The reduction in Lp(a) levels reached a peak of 98% with the 600 mg dose of SLN360 (30 mg: −46%, 100 mg: −86%, 300 mg: −96%, 600 mg: −98%, placebo: −10%), and the reduced levels were maintained until day 150 with adequate safety [47]. In the phase 2 clinical multicenter trial in patients with stable ASCVD with serum lipoprotein(a) concentrations greater than or equal to 125 nmol/L at 26 sites in Europe and South Africa, compared to the placebo group, Zerlasiran was well-tolerated with mean time-averaged percent change in lipoprotein(a) concentration from baseline to week 36 was − 85.6% (95% CI, − 90.9% to − 80.3%), − 82.8% (95% CI, − 88.2% to − 77.4%), and − 81.3% (95% CI, − 86.7% to − 76.0%) for the 450 mg every 24 weeks, 300 mg every 16 weeks, and 300 mg every 24 weeks groups, respectively [48]. Zerlasiran demonstrated Lp(a) reductions exceeding 90% at the 300 mg and 600 mg doses, with reductions persisting through 60 weeks of follow-up after a single injection, and no safety concerns [48].

Lepodisiran (formerly LY3819469), developed by Eli Lilly, is also a GalNac siRNA with a unique tetraloop structure, designed to achieve lasting reductions in Lp(a) synthesis by targeting the production of apo(a) by the liver [49]. In a phase 1 clinical trial conducted in the United States and Singapore, 48 subjects without CVD and with Lp(a) levels exceeding 30 mg/dL were enrolled [49]. Participants were randomized to receive either a placebo or a single subcutaneous dose of lepodisiran (4 mg, 12 mg, 32 mg, 96 mg, 304 mg, and 608 mg). Lepodisiran led to a dose-dependent decrease in serum Lp(a) levels, with a maximal median change exceeding the 90% observed for the three highest doses studied (96, 304, and 608 mg) [49]. In the Phase 2 (ALPACA) trial (NCT05565742), 320 participants across 66 centers in 12 countries were enrolled, with a median baseline concentration of 253.9 nmol/L [50]. The study evaluated five dosing regimens: 16 mg, 96 mg, or 400 mg of lepodisiran at baseline and day 180; 400 mg at baseline with a placebo at day 180; and a double placebo group [50]. The primary endpoint, assessing the placebo-adjusted, mean change in Lp(a) from days 60 to 180, showed reductions of − 40.8% (95% CI, − 55.8 to − 20.6) for 16 mg, − 75.2% (95% CI, − 80.4 to − 68.5) for 96 mg, and − 93.9% (95% CI, − 95.1 to − 92.5) for the pooled 400 mg group [50]. At one year, Lp(a) levels remained 91% below baseline in the double-dose 400 mg group; at 18 months, they were 74% below baseline after a single 400 mg injection [50]. These findings strongly suggest that once-yearly dosing may be clinically feasible. The ongoing ACCLAIM-Lp(a) phase 3 trial (NCT06292013) is projected to be the largest Lp(a) outcomes trial in history, with an estimated enrollment of 17,300 participants across 932 locations [51]. Its design is uniquely ambitious: unlike HORIZON and OCEAN(a), it includes both secondary prevention (established ASCVD) and high-risk primary prevention populations [51]. The inclusion threshold is set at Lp(a) ≥ 175 nmol/L, with the primary endpoint being the time to the first 4-point MACE. The study is expected to be completed by 2029 [51]. This trial has the potential to reshape cardiovascular prevention guidelines beyond the ASCVD context.

#### Small Molecule Inhibitors

Muvalaplin (formerly LY3473329) is a first-in-class small molecule developed to inhibit the formation of Lp(a) particles, representing the first oral agent specifically developed to reduce serum Lp(a) levels [52]. The main mechanism underlying muvalaplin’s function is its potential similarity to naturally occurring variants in apo(a) that cannot interact with ApoB100 [52]. Instead of targeting apo(a) mRNA, it prevents Lp(a) particle assembly by blocking the initial non-covalent interaction between a specific apo(a) region (the KIV-7 and KIV-8 kringle domains) and ApoB-100 on LDL-like particles [53, 54]. This interruption stops disulfide bond formation and prevents Lp(a) formation [53].

Recent findings from a phase 1 randomized, placebo-controlled human trial by Nicholls et al. assessed muvalaplin’s effects on serum Lp(a) levels [53]. After dividing into two groups, one group received a single ascending dose of muvalaplin (ranging from 1 mg to 800 mg), while the second group, comprising participants with elevated Lp(a) concentrations above 30 mg/dL, received multiple single daily doses of muvalaplin (30–800 mg) for a two-week period [53]. The primary outcomes assessed were safety, tolerability, pharmacokinetic, and pharmacodynamic parameters. The drug led to a rapid reduction in serum Lp(a) levels within 1 day, with further decreases after repeated dosing in a dose-dependent manner, reaching up to 65% with a daily oral regimen for 2 weeks [53]. Notably, the maximum reduction in Lp (a) levels was observed on days 14 and 15, and these reductions persisted for up to 50 days after the last dose, especially for doses ≥ 300 mg [53]. Muvalaplin did not interfere with plasminogen or cause significant changes in other lipid parameters [53]. In the phase 2 KRAKEN trial (NCT05563246), which enrolled 233 adults with Lp(a) > 175 nmol/L and high cardiovascular risk at 43 sites across Asia, Europe, Australia, Brazil, and the US, were randomized to receive treatment with placebo or muvalaplin at dosages of 10 mg/d, 60 mg/d, or 240 mg/d for 12 weeks [55]. Muvalaplin produced placebo-adjusted reductions in lipoprotein(a) by up to − 47.6% (95% CI, 35.1–57.7%) with 10 mg/d, − 81.7% (95% CI, 78.1–84.6%) with 60 mg/d, and − 85.8% (95% CI, 83.1–88.0%) with 240 mg/d using an intact lipoprotein(a) assay and by up to − 40.0% to 70.0% using an apolipoprotein(a) assay, without increase in clinically significant adverse events [55].

To assess the impact of muvalaplin on MACE, an ongoing MOVE-Lp(a) phase 3 trial targeting 10,450 patients began in late 2025 and is expected to be completed by 2031 [56]. If successful, muvalaplin would be the first oral Lp(a)-lowering therapy, marking a paradigm shift for potentially hundreds of millions of patients globally who require treatment.

#### Other Medications Currently in Development

Obicetrapib is an oral cholesteryl ester transfer protein (CETP) inhibitor developed by NewAmsterdam Pharma that is tested as an adjunct to high-intensity statin therapy in patients with dyslipidemia [57]. Although not primarily designed to reduce Lp(a), the ROSE1 and ROSE2 Phase 2 trials showed that, compared with placebo, obicetrapib reduced Lp(a) by more than 57% [57]. This was a significant off-target benefit, in addition to its primary LDL-lowering effect [57]. Hence, offering promising potential for ASCVD management. The ongoing VINCENT trial (NCT06496243), launched in December 2024, primarily evaluates the impact of obicetrapib on Lp(a) reduction and is expected to be completed by 2027 [58].

More recently, the phase 3 BROOKLYN trial in patients with heterozygous familial hypercholesterolemia reported that obicetrapib produced a placebo-adjusted Lp(a) reduction of 45.9% at day 84, an effect that persisted through one year [59]. The larger phase 3 BROADWAY trial, which enrolled patients with established ASCVD and/or heterozygous familial hypercholesterolemia, corroborated this off-target Lp(a)-lowering effect alongside its primary LDL-C benefit in a considerably larger and more clinically diverse population than the earlier ROSE1/ROSE2 analyses [60]. A pooled analysis of the BROOKLYN and BROADWAY trials (*n* = 2356) examined how obicetrapib affects Lp(a), showing overall placebo-adjusted reductions of 37.3% and 14.9 nmol/L [61]. Similar to what has been seen with PCSK9 inhibitors, the percentage reduction was higher in patients with baseline Lp(a) levels of 50 to less than 150 nmol/L (43.3%) compared to those with levels of 150 nmol/L or more (12.6%) [61]. However, the absolute reduction was comparable between these groups (36.3 vs. 32.3 nmol/L) [61]. This is clinically important because most RNA-targeted Lp(a)-lowering drugs require higher initial Lp(a) levels (150–175 nmol/L or more) for trial inclusion, possibly excluding patients with moderate Lp(a) elevations from treatment [61]. The similar absolute reduction across the range suggests obicetrapib might help fill this treatment gap, subject to confirmation of cardiovascular benefits. Notably, the analysis included many patients with normal Lp(a) levels at baseline, so further studies focusing on populations with high Lp(a) are necessary.

#### Genomic Editing

Beyond RNA therapeutics, another mechanistic class, through permanent genomic editing, has entered clinical trials.

CTX320 (CRISPR Therapeutics) uses an in vivo CRISPR/Cas9 gene-editing therapy delivered via Lipid Nanoparticles (LNPs) that directly targets and disrupts the LPA gene in hepatocytes [62]. CTX320 uses canonical Cas9 mRNA and a guide RNA designed to create a permanent reduction in apo(a) production with lifelong benefit from a single IV infusion [62]. The Phase 1 trial in patients with elevated Lp(a) and ASCVD or aortic valve stenosis, with results expected in the first half of 2026 [62]. This could potentially be a one-time treatment and represents the most futuristic approach.

### Comparative Safety Profile

Earlier generations of siRNA medications had safety concerns, including peripheral neuropathy and an unexplained high mortality [30]. The chemical modifications incorporated into olpasiran, as mentioned earlier, were implemented to address these potential risks and stabilize the duplex against nucleolytic degradation [30]. Also, conjugation with GalNAc enables olpasiran to achieve specific, efficient delivery to hepatocytes [30]. These improvements in siRNA technology enable subcutaneous administration at lower doses, with improved biodistribution and extended duration of action compared with previous lipid nanoparticle formulations [30] (Table 2). These modifications also led to favorable safety profiles.

**Table 2:** Comparative efficacy of the novel Lp(a)-lowering therapies

Agent	Class	Route	Frequency	Max Lp(a) reduction	Key feature
Pelacarsen	ASO	SC injection	Monthly	~ 80%	First mover; pivotal CVOT data imminent
Olpasiran	siRNA	SC injection	Every 12 weeks	> 95%	Prolonged off-treatment effect; OxPL reduction
Lepodisiran	siRNA	SC injection	~Yearly	~ 94–97%	Extraordinary durability; once-yearly potentially feasible
Zerlasiran	siRNA	SC injection	~Every 16 weeks	~ 96%	Cumulative stacking effect
Muvalaplin	Small molecule	Oral daily	Daily	~ 86% (intact assay)	First oral agent
CTX320	CRISPR/Cas9	IV infusion (once)	Once only	Ongoing	Permanent genomic edit; “one and done” potential

Lp*(a) *Lipoprotein (a), *ASO *Antisense oligonucleotides, *siRNA *Small interfering RNA, *SC *Subcutaneous, *IV *Intravenous, *CVOT *Cardiovascular outcomes trials, *CRISPR *Clustered regularly interspaced short palindromic repeats, *OxPL *Oxidized phospholipids

Pelacarsen: Tsimikas et al. noted no significant differences between the pelacarsen and placebo groups in adverse events (90% versus 87%, respectively), although 10% of the pelacarsen group reported serious adverse events, compared with 2% with placebo [32]. The reported adverse events included myalgia, influenza-like symptoms, and urinary tract infections [32]. Reported adverse events included myalgia, influenza-like symptoms, and urinary tract infections; there were no significant, dose-dependent differences between pelacarsen and placebo in platelet count or in hepatic and renal function [32]. Injection-site reactions were noted in 27% of patients who received pelacarsen, with erythema being the most common reaction [32].

Olpasiran: In the OCEAN(a)-DOSE trial, the overall incidence of adverse events, serious adverse events, and treatment discontinuations was similar between olpasiran and placebo, including a 2% discontinuation rate in each group [40]. Injection-site reactions, primarily mild pain, were the most common olpasiran-related adverse event and occurred somewhat more often at higher doses, but were transient and infrequently led to discontinuation [39, 40]. Extended off-treatment follow-up approaching one year revealed no new safety signals, with hepatic, renal, and hematologic parameters comparable across groups [41].

Zerlasiran: Zerlasiran was well tolerated through 36 weeks of follow-up, with injection-site pain, the most common treatment-related adverse event, occurring in only 2.3% to 7.1% of participants on the first day after dosing [48]. Twenty serious adverse events occurred in 17 patients, none considered related to the study drug [48].

Muvalaplin: As an oral agent, muvalaplin avoids injection-site reactions altogether. In the KRAKEN trial, overall adverse event rates were similar between the muvalaplin and placebo arms, with no significant excess of treatment-related events, although discontinuation was somewhat more frequent in the highest-dose arm (240 mg) [55].

Obicetrapib: Obicetrapib has consistently shown an excellent safety and tolerability profile, from the earlier pooled analysis of the ROSE1 and ROSE2 trials, through the considerably larger phase 3 BROOKLYN and BROADWAY trials [57, 59, 60, 63]. In BROOKLYN, the treatment discontinuation rate was lower with obicetrapib than with placebo (7.6% versus 14.4%), with no increase in blood pressure and safety results otherwise comparable to placebo [59]. BROADWAY corroborated this favorable safety profile in a considerably larger and higher-risk population [60]. 

CTX320: As a single-dose, in vivo CRISPR/Cas9 gene-editing therapy still in early Phase 1 testing, comprehensive comparative safety data for CTX320 are not yet available. Based on class experience with lipid nanoparticle-delivered gene-editing agents in this pipeline, particular attention will be needed to infusion-related reactions and hepatic safety parameters as longer-term data mature.

### Current Guidelines, Recommendations, and Clinical Implications

Recent guidelines now reflect a clear shift toward routine assessment. The 2022 European Atherosclerosis Society (EAS) consensus statement and the 2024 National Lipid Association (NLA) updates mark a shift toward more aggressive screening strategies [4, 64]. These organizations recommend that every adult should have their Lp(a) concentration measured at least once in their lifetime [4, 64]. This recommendation is echoed by the 2026 ACC/AHA guidelines on management of patients with dyslipidemia, which emphasize that early identification is crucial because Lp(a) levels are largely determined by genetics and remain relatively stable throughout life [65, 66]. Hence, a single measurement is typically sufficient for lifelong risk stratification [67]. Furthermore, cascade testing is advised for families with a known history of very high Lp(a) levels [64, 68]. Secondary causes of elevated Lp(a), including kidney, liver, or thyroid disease, pregnancy, menopause, and select medications, should be considered when interpreting results [68, 69]. Despite these recommendations, testing remains underutilized, especially in older adults and Black individuals.

The latest ACC/AHA 2026 guideline recommendations place lipoprotein(a) (Lp(a)) firmly into routine cardiovascular risk assessment, issuing multiple Class 1 recommendations that standardize its clinical use [65]. In all adults, a one-time measurement of Lp(a) is now recommended for atherosclerotic cardiovascular disease (ASCVD) risk stratification, reflecting its role as a genetically determined risk enhancer [65]. In patients with familial hypercholesterolemia, premature ASCVD, or known elevated Lp(a), cascade testing of first-degree relatives is recommended to identify additional individuals at increased risk [62, 66]. For those with elevated Lp(a) (≥ 125 nmol/L or ≥ 50 mg/dL), aggressive and early optimization of all modifiable cardiovascular risk factors is advised to mitigate downstream ASCVD risk [70]. In patients with established ASCVD and elevated Lp(a) who have not reached LDL-C or non-HDL-C targets despite maximally tolerated statin therapy, the addition of a PCSK9 monoclonal antibody with proven cardiovascular benefit is recommended to further reduce risk [65]. Together, these Class 1 recommendations establish Lp(a) as a routinely assessed and clinically actionable biomarker in modern preventive cardiology.

Implementing universal Lp(a) screening is transforming cardiovascular prevention by revealing risk factors that clinical features alone cannot predict. Contemporary guidelines emphasize its role as a “risk enhancer,” identifying a subset (~ 10–20%) of individuals at substantially higher ASCVD risk [65]. Clinically, Lp(a) should be measured at least once in adulthood, with standardized reporting in nmol/L when available [64, 71]. In patients with elevated levels, clinicians should intensify overall risk-factor management and consider lower LDL-C targets, particularly in secondary-prevention populations [4, 64]. Cascade screening of relatives remains essential for early identification of high-risk individuals. Overall, while no Lp(a)-specific therapies are yet part of routine care, guideline-directed intensive risk factor modification remains the cornerstone of management and substantially mitigates excess cardiovascular risk.

### Limitations

This review has several limitations. First, with the exception of pelacarsen, olpasiran, lepodisiran, and obicetrapib, most discussed agents have only undergone early-phase trials, and none of the Lp(a)-lowering therapies have yet proven to reduce cardiovascular events. The ongoing outcome trials (Lp(a)HORIZON, OCEAN(a)-OUTCOMES, ACCLAIM-Lp(a)) are necessary to determine if pharmacologically lowering Lp(a) translates into clinical benefits. Throughout this review, discussion of these agents should be viewed as mechanistic and biomarker evidence, rather than direct proof of cardiovascular efficacy. Second, efficacy and safety data for individual agents come from separate, non-comparative trials that differ significantly in patient populations, background therapies, Lp(a) assay methods, doses, and follow-up periods. Therefore, any cross-agent comparisons, such as in Table 2 and the Comparative Safety Profile section, are meant to be indirect and hypothesis-generating rather than definitive rankings of efficacy or safety. Third, the high Lp(a) thresholds (150–175 nmol/L or more) used for enrollment in most outcomes trials may limit the applicability of these findings to the broader patient population with moderately elevated Lp(a). Fourth, long-term safety data beyond 1–2 years are limited for all agents, especially for genomic editing approaches, which are still in early Phase 1 testing. Finally, this is a narrative rather than a systematic review, without a formal search protocol or risk-of-bias assessment.

## Conclusion

The Lp(a) therapeutic landscape is rapidly evolving from theoretical promise into a clinically developed pipeline on the cusp of regulatory approval, with five distinct drug classes now in clinical development: three injectable nucleic acid-based agents in Phase 3 cardiovascular outcomes trials, one oral small-molecule inhibitor in Phase 2/3, and gene-editing approaches entering early clinical testing [33, 43, 51, 57, 62]. No Lp(a)-specific therapy is yet approved, but results from the first outcomes trials are expected by mid-to-late 2026 [33, 43]. Three agents targeting hepatic apo(a) mRNA production are furthest along in development, all conjugated to GalNAc for hepatocyte-specific delivery: Pelacarsen (antisense oligonucleotide), Olpasiran (small interfering RNA (siRNA)), and Lepodisiran (siRNA). Together, these will either support or refute the Lp(a) hypothesis and clarify if Lp(a)-lowering should become as central as LDL-lowering in cardiovascular prevention. Gene editing represents a potential one-time, permanent solution for elevated Lp(a). A 2026 ACC Scientific Statement on gene editing in cardiovascular disease highlighted several programs now entering clinical development, including CTX320 (CRISPR Therapeutics), VERVE-301 (Verve Therapeutics), and cytosine base editing [72]. Collectively, these developments signal a move toward targeted, long-lasting, and possibly one-time therapies for Lp(a) reduction, with upcoming outcomes trials set to define their clinical impact (Central Illustration).

## Data Availability

No datasets were generated or analysed during the current study.

## Key References

- Tsimikas S, Marcovina SM. Ancestry, Lipoprotein(a), and Cardiovascular Risk Thresholds: JACC Review Topic of the Week. J Am Coll Cardiol. 2022;80(9):934–946. 10.1016/j.jacc.2022.06.019
  - *Establishes ancestry-specific Lp(a) risk thresholds and highlights how population-level differences in Lp(a) distribution affect risk interpretation*,* directly underpinning the risk-threshold values and ancestry discussion in the Introduction.*
- Nordestgaard BG, Langsted A. Lipoprotein(a) and cardiovascular disease. Lancet. 2024;404(10459):1255–1264. 10.1016/S0140-6736(24)01308-4
  - *Comprehensive contemporary review establishing the epidemiological and ancestry-based distribution of Lp(a)*,* which underpins the risk-stratification framework used throughout this review.*
- Greco A, Finocchiaro S, Spagnolo M, et al. Lipoprotein(a) as a Pharmacological Target: Premises, Promises, and Prospects. Circulation. 2025;151(6):400–415. 10.1161/CIRCULATIONAHA.124.069210
  - *Synthesizes the mechanistic rationale for Lp(a)-lowering therapy and situates the current drug pipeline within the broader cardiovascular prevention landscape.*
- Novartis Pharmaceuticals. A Randomized Double-Blind, Placebo-Controlled, Multicenter Trial Assessing the Impact of Lipoprotein (a) Lowering With Pelacarsen (TQJ230) on Major Cardiovascular Events in Patients With Established Cardiovascular Disease [Lp(a)HORIZON]. clinicaltrials.gov; 2026. Accessed June 30, 2026. https://clinicaltrials.gov/study/NCT04023552
  - *The largest and most highly anticipated Lp(a) cardiovascular outcomes trial worldwide; as the first pivotal phase 3 study testing whether pharmacological Lp(a) lowering with pelacarsen reduces major cardiovascular events*,* its results (expected mid-to-late 2026) will either establish or refute the Lp(a)-lowering hypothesis and are poised to reshape cardiovascular prevention guidelines.*
- Nissen SE, Wang Q, Nicholls SJ, et al. Zerlasiran-A Small-Interfering RNA Targeting Lipoprotein(a): A Phase 2 Randomized Clinical Trial. JAMA. 2024;332(23):1992–2002. 10.1001/jama.2024.21957
  - *Pivotal phase 2 randomized trial demonstrating sustained, dose-dependent Lp(a) reductions exceeding 80% with zerlasiran, directly informing the comparative efficacy data summarized in Table *2.
- Nissen SE, Ni W, Shen X, et al. Lepodisiran - A Long-Duration Small Interfering RNA Targeting Lipoprotein(a). N Engl J Med. 2025;392(17):1673–1683. 10.1056/NEJMoa2415818
  - *Landmark New England Journal of Medicine trial establishing the durability of lepodisiran’s Lp(a)-lowering effect*,* supporting the feasibility of once-yearly dosing and the design of the ongoing ACCLAIM-Lp(a) outcomes trial.*
- Nicholls SJ, Nissen SE, Fleming C, et al. Muvalaplin, an Oral Small Molecule Inhibitor of Lipoprotein(a) Formation: A Randomized Clinical Trial. JAMA. 2023;330(11):1042–1053. 10.1001/jama.2023.16503
  - *First-in-human randomized trial of muvalaplin*,* the first oral Lp(a)-lowering agent*,* providing the foundational safety and pharmacodynamic data that underlie the KRAKEN and MOVE-Lp(a) trial programs.*
- Diaz N, Perez C, Escribano AM, et al. Discovery of potent small-molecule inhibitors of lipoprotein(a) formation. Nature. 2024;629(8013):945–950. 10.1038/s41586-024-07387-z
  - *Defines the structural basis by which small molecules block apo(a)-ApoB100 assembly*,* providing the mechanistic foundation for the oral small-molecule inhibitor class discussed in this review.*
- Blumenthal RS, Morris PB, Gaudino M, et al. 2026 ACC/AHA/AACVPR/ABC/ACPM/ADA/AGS/APhA/ASPC/NLA/PCNA Guideline on the Management of Dyslipidemia: A Report of the American College of Cardiology/American Heart Association Joint Committee on Clinical Practice Guidelines. J Am Coll Cardiol. 2026;87(19):2624–2757. 10.1016/j.jacc.2025.11.016
  - *The current*,* authoritative U.S. guideline issuing Class 1 recommendations for universal one-time Lp(a) screening and cascade testing*,* forming the clinical-practice backbone of the guidelines section of this review.*
- Gene Editing Therapy in Cardiovascular Disease: 2026 ACC Scientific Statement: A Report of the American College of Cardiology. J Am Coll Cardiol. Accessed June 30, 2026.https://www.jacc.org/. 10.1016/j.jacc.2026.02.5092
  - *Most recent scientific statement cataloging CRISPR/Cas9 and base-editing programs targeting LPA*,* framing the genomic-editing horizon discussed in the conclusion.*
